# Evaluation of the BD Phoenix CPO Detect Panel for Detection and Classification of Carbapenemase Producing *Enterobacterales*

**DOI:** 10.3390/antibiotics12071215

**Published:** 2023-07-21

**Authors:** Harshad Lade, Seri Jeong, Kibum Jeon, Han-Sung Kim, Hyun Soo Kim, Wonkeun Song, Jae-Seok Kim

**Affiliations:** 1 Department of Laboratory Medicine, Hallym University College of Medicine, Kangdong Sacred Heart Hospital, Seoul 05355, Republic of Korea; harshadlade@hallym.ac.kr; 2 Department of Laboratory Medicine, Hallym University College of Medicine, Kangnam Sacred Heart Hospital, Seoul 07441, Republic of Korea; 3 Department of Laboratory Medicine, Hallym University College of Medicine, Hangang Sacred Heart Hospital, Seoul 07247, Republic of Korea; 4 Department of Laboratory Medicine, Hallym University College of Medicine, Hallym University Sacred Heart Hospital, Anyang 14068, Republic of Korea; 5 Department of Laboratory Medicine, Hallym University College of Medicine, Dongtan Sacred Heart Hospital, Hwaseong 18450, Republic of Korea

**Keywords:** carbapenem-resistant *Enterobacterales*, carbapenemase producing organisms, carbapenemase detection, Amber classification, Phoenix NMIC-500, CPO detect panel

## Abstract

Carbapenem-resistant *Enterobacterales* (CRE) pose a serious public health threat due to their resistance to most antibiotics. Rapid and correct detection of carbapenemase producing organisms (CPOs) can help inform clinician decision making on antibiotic therapy. The BD Phoenix™ CPO detect panel, as part of antimicrobial susceptibility testing (AST), detects carbapenemase activity (P/N) and categorizes CPOs according to Ambler classes. We evaluated a CPO detect panel against 109 carbapenemase producing *Enterobacterales* (CPE) clinical isolates from Korea. The panel correctly detected carbapenemases production in 98.2% (*n* = 107/109) isolates and identified 78.8% (*n* = 26/33) class A, 65.9% (*n* = 29/44) class B, and 56.3% (*n* = 18/32) class D carbapenemase producers as harboring their corresponding Ambler classes. Specifically, the panel correctly classified 81.3% (*n* = 13/16) of *K. pneumoniae* KPC isolates to class A. However, the panel failed to classify 40.0% (*n* = 4/10) IMP and 63.6% (*n* = 7/11) VIM isolates to class B. Despite 27.5% (*n* = 30/109) CPE not being assigned Ambler classes, all of them tested carbapenemase positive. Our results demonstrate that the CPO detect panel is a sensitive test for detecting CPE and classifying KPC as class A, helping with antibiotics selection, but one-third of CPE remained unclassified for Ambler classes.

## 1. Introduction

Carbapenem-resistant *Enterobacterales* (CRE) are Gram-negative bacteria defined by the United States Centers for Disease Control and Prevention (CDC) as *Enterobacterales* (formerly *Enterobacteriaceae*) that are typically resistant to at least one carbapenem antibiotic (imipenem, meropenem, ertapenem, and doripenem) [1]. *Enterobacterales* (such as *Citrobacter freundii, Escherichia coli, Enterobacter aerogenes, Enterobacter cloacae, Klebsiella oxytoca, Klebsiella pneumoniae*, and *Serratia marcescens*) are commonly found in the human gastrointestinal tract, causing various infectious diseases such as urinary tract infection, pneumonia, pyelonephritis, meningitis, and sepsis [2]. Carbapenems have long been considered the “last resort” antibiotics for treating patients with severe infections caused by *Enterobacterales* [3], but the effectiveness of this class of antibiotics is threatened by the emergence and spread of CRE [4,5]. Moreover, CRE is often resistant to multiple or all antibiotics, leaving limited or no effective treatment options [6,7]. In the early 1990s, CRE strains (*S. marcescens*) were found in Japan and subsequently in neighboring countries [8]. The first case of CRE (KPC-2 producing *K. pneumoniae*) was reported in 2010 in Korea [9], and later the number of CRE infections increased significantly from 5717 in 2017 to 15,369 in 2019 [10].

Carbapenem resistance in *Enterobacterales* is most frequently mediated by one of two principal mechanisms. First, the hyperproduction of extended-spectrum β-lactamases (ESBLs) or AmpC cephalosporinases (such as AmpC β-lactamase) [3], especially in combination with decreased cell wall permeability to carbapenems or increased efflux pump activity due to simultaneous accumulation of mutations in genes coding outer membrane porins (such as *OmpK35* and *OmpK36*) [11,12,13]. Second, the production of carbapenemase enzymes [14,15], which hydrolyze carbapenems by so-called carbapenemase producing *Enterobacterales* (CPE). Distinguishing between these two resistance mechanisms can provide valuable insights and guidance in terms of infection control. In the United States, carbapenemase producing organisms (CPOs) accounted for 59% of 1040 patients with CRE infections in a multicenter cohort study from April 2016 to August 2017 [16].

Carbapenemases identified in *Enterobacterales* are categorized into three molecular classes based on their amino acid sequences according to the Ambler classification scheme [17,18]. These are (i) Ambler class A (*K. pneumoniae* carbapenemase (KPC)) [19], (ii) Ambler class B or metallo-β-lactamases (MBL) (Verona integron-borne metallo-β-lactamase (VIM), New Delhi metallo-β-lactamase (NDM), and to lesser extent imipenemase-type metallo-β-lactamas (IMP)) [5,20], and (iii) Ambler class D (OXA-48-like in *K. pneumoniae*) [21,22,23]. The carbapenemases-encoding genes are often located on mobile genetic elements (MGEs) such as plasmids, insertion sequences, integrons, and transposons [24], leading to further spread. These MGEs also carry additional genes conferring resistance to multiple other antibiotics, such as fluoroquinolones and aminoglycosides, thereby limiting the treatment options possibly active against CPE [25,26].

In the last decade, different newer agents which combine β-lactam plus β-lactamase inhibitors have been developed against certain carbapenem-resistant Gram-negative bacteria [27]. They include ceftazidime–avibactam, ceftolozane–tazobactam, meropenem–vaborbactam, imipenem–relebactam, cefiderocol, eravacycline, and plazomicin. The Infectious Diseases Society of America (IDSA) [28,29] and the European Society of Clinical Microbiology and Infectious Diseases (ESCMID) [30] recommend ceftazidime–avibactam, meropenem–vaborbactam, and imipenem–relebactam for treatment of severe infections caused by CRE [31]. Therefore, distinguishing carbapenemase producing CRE from non-producers is important to prioritize newer agents for patients infected with CRE and prevent indiscriminate use that may lead to resistance emergence [32].

The BD Phoenix™ CPO detect panel (Becton Dickinson Diagnostic Systems, Sparks, MD, USA) is a bacterial growth-based antimicrobial susceptibility testing (AST) assay that detects carbapenemase activity (P/N) in *Enterobacterales*, *Pseudomonas aeruginosa*, and *Acinetobacter baumannii* and classifies carbapenemase producers to Ambler class A, class B, and class D in 6–16 h, when used with the Phoenix™ M50 system. To detect and classify carbapenemases, the CPO detect panel uses meropenem, doripenem, temocillin, and cloxacillin, either separately or in combination with various chelators and β-lactamase inhibitors [33]. An earlier study that evaluated the BD Phoenix NMIC-500 panel against 47 CPE found a 97.9% sensitivity for carbapenemase detection [34].

To ensure the sensitivity and reliability of the BD Phoenix CPO detect panel over time and across different demographic regions, it is necessary to evaluate its performance against CPE clinical isolates that are in line with current local epidemiology. Here, we evaluated the BD CPO detect panel to correctly detect and classify carbapenemases of Ambler class A, B, and D against a characterized set of CPE clinical isolates in Korea.

## 2. Results

### 2.1. CPE Isolates Characteristics

**Figure 1:**
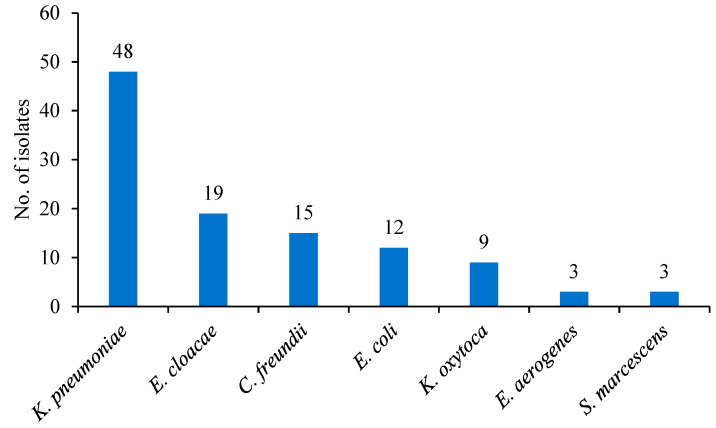
Distribution of selected CPE clinical isolates by species (*n* = 109).

All *Enterobacterales* clinical isolates used in this study were positive for carbapenemase genes by PCR, thus referred to as CPE henceforth. Figure 1 shows the distribution of the 109 CPE isolates by species. Among the CPE clinical isolates tested (*n* = 109), *K. pneumoniae (n* = 48) accounted for the highest proportion, followed by *E. cloacae (n* = 19), *C. freundii (n* = 15), *E. coli (n* = 12), *K. oxytoca (n* = 9), *E. aerogenes (n* = 3), and *S. marcescens (n* = 3).

Carbapenemases belonging to three Ambler classes, namely class A, class B, and class D, were identified by PCR. The PCR and Sanger sequencing identified distinct carbapenemase subtypes, and Ambler classes are shown in Table 1. The genotypic characterization of the 109 CPE clinical isolates revealed the presence of 33 class A carbapenemases (KPC, *n* = 33), 44 class B carbapenemases (IMP, *n* = 10; NDM, *n* = 23; VIM, *n* = 11), and 32 class D carbapenemases (OXA-48-like, *n* = 32) producers. Among the predominant *K. pneumoniae* isolates (*n* = 48), there were 16 strains of KPC class A carbapenemase, 12 strains of class B (3 IMP, 5 NDM, 4 VIM), and 20 strains of OXA-48-like class D carbapenemase producers.

### 2.2. Carbapenemase Detection and Ambler Classification by BD CPO Detect Panel

The BD CPO detect panel was evaluated for the detection of carbapenemase and its Ambler classification in CPE clinical isolates. BD CPO detect panel results were considered carbapenemase positive if they agreed with the presence of carbapenemase genes as characterized by PCR. Among 109 CPE, the CPO detect panel correctly detected carbapenemase production in 98.2% (*n* = 107/109) isolates (Table 1). The *C. freundii* isolate producing OXA-48-like class D carbapenemase was identified as carbapenemase-negative, whereas one *K. oxytoca* isolate producing VIM class B carbapenemase was identified as an ESBL producer by the BD CPO detect panel. Further retesting of VIM class B carbapenemase producing *K. oxytoca* strain by duplicated BD CPO detect panel showed discrepancies in categorization to class B (Supplementary Materials Table S1). The only one carbapenemase-negative *C. freundii* was found sensitive to ertapenem (0.5 and ≤0.25 μg/mL) and meropenem (≤0.25 and ≤0.25 μg/mL), while intermediate to imipenem (2 and 2 μg/mL), when retested using the BD CPO detect panel (Table S2). The *K. oxytoca* strain showed a discrepancy in the carbapenem susceptibility pattern when retested by the BD CPO detect panel (Table S2). Furthermore, duplicate MicroScan tests showed the discrepancy in the carbapenem susceptibility pattern of this VIM class B carbapenemase producing *K. oxytoca* (Table S2).

Overall, the BD CPO detect panel correctly identified 67.0% (*n* = 73/109) clinical isolates (including 26 KPC, 6 IMP, 20 NDM, 3 VIM, and 18 OXA-48-like carbapenemases producers) as harboring their corresponding Ambler classes (Table 1). Specifically, correct Ambler classification was observed for 78.8% (*n* = 26/33) of class A, 65.9% (*n* = 29/44) of class B, and 56.3% (*n* = 18/32) of class D carbapenemase producers. The CPO detect panel showed the highest sensitivity in identifying KPC class A carbapenemases producers, but 6.1% (*n* = 2/33) strains were incorrectly classified as class B carbapenemase producers. Importantly, the BD CPO detect panel correctly identified 81.3% (*n* = 13/16) of *K. pneumoniae* KPC isolates as harboring class A carbapenemase, which are dominant strains in this class. For Amber class B, the CPO detect panel correctly classified 87.0% (*n* = 20/23) NDM, followed by 60.0% (*n* = 6/10) IMP, and 27.3% (*n* = 3/11) VIM isolates. No class B carbapenemase producer strains were incorrectly classified to other classes by the CPO detect panel. One *K. oxytoca* strain producing VIM class B carbapenemase showed ESBL production (Table S1). The sensitivity in the identification of class D OXA-48-like carbapenemase producers was 56.3% (*n* = 18/32), but 6.25% (*n* = 2/32) isolates (*K. oxytoca* and *K. pneumoniae*) were incorrectly identified as class B carbapenemase producers. Of the 20 *K. pneumoniae* OXA-48-like isolates, the CPO detect panel correctly identified 15 as harboring class D carbapenemase, while 1 was misclassified as a class B carbapenemase producer and 4 remained as unclassified to any Ambler class.

A total of 27.5% (*n* = 30/109) *Enterobacterale* isolates identified as “carbapenemase producers” by the BD CPO panel were unclassified to Ambler class A, class B, or class D. Specifically, 15.2% (*n* = 5/33) of class A, 31.8% (*n* = 14/44) of class B, and 34.4% (*n* = 11/33) of class D carbapenemase producers were unclassified for Ambler class. The BD CPO detect panel flagged the presence of a carbapenemase without further Ambler classification in 13 *K. pneumoniae*, 7 *E. cloacae*, 1 *C. freundii*, 3 *E. coli*, 4 *K. oxytoca*, and 2 *S. marcescens* isolates.

## 3. Discussion

In recent years, there has been a significant increase in CRE infections in many countries worldwide. CRE caused 13,100 infections in the United States in 2017 and 68,000 infections in Europe [35,36]. The increasing importance of CRE infections necessitates the methods for rapid detection of CPOs and classification of carbapenemase according to Ambler classes. Rapid and correct detection of CPOs and specification of carbapenemases into the various Ambler classes can assist clinicians in making decisions on appropriate patient treatment and infection control measures to prevent outbreaks in clinical settings [37]. The BD Phoenix™ CPO detect panel is an automated platform that allows carbapenem susceptibility testing and detects carbapenemase producers and classifies them to Ambler class A, class B, and class D. Here, we evaluated the BD CPO detect panel for detecting carbapenemase production (P/N) in 109 CPE clinical isolates from five different hospitals and categorize them to Ambler class A, class B, and class D.

The results showed that the BD CPO detect panel detected carbapenemase production in 98.2% of CPE clinical isolates, while classifying only 67.0% of isolates to their corresponding Ambler classes. The detection rate of CPE by the BD CPO detect panel was high except for one OXA-48-like class D producing *C. freundii*, which was sensitive to ertapenem and meropenem but intermediate to imipenem. Our overall analytical performance for carbapenemase detection in CPE clinical isolates was consistent with those reported by Simon and co-workers [38] and with Cho and co-workers [34], where the sensitivity was 100% and 97.9%, respectively. In a study by Simon et al., 57 CPE isolates were tested with the BD Phoenix CPO detect panel, which resulted in a sensitivity of 100% in detecting carbapenemase production [38]. Cho et al. evaluated the performance of the BD Phoenix NMIC-500 test with 47 CPE isolates and reported a sensitivity of 97.9% in detecting CPOs [34]. Both the previous reports and our results demonstrate the high sensitivity of the BD CPO detect panel for CPE detection. Our findings suggest that the BD CPO detect panel’s positive results for carbapenemase detection (P/N) can be reported as CRE and requires no additional confirmatory test. However, one VIM class B carbapenemase producing *K. oxytoca* isolate detected as an ESBL producer by the CPO detect panel was suspicious for carbapenem resistance and required confirmation through an additional AST method.

The discrimination between Ambler classes provides useful information for the treatment of CRE infections with novel β-lactam plus β-lactamase inhibitor combinations [31,39]. Most of such novel agents exhibited in vitro activity against various β-lactamases of Ambler class A, class B, and class D; however, ceftazidime–avibactam, ceftolozane–tazobactam, and meropenem–vaborbactam were found to be inactive against MBL [40]. Flora Cruz-López et al. described ceftazidime–avibactam as active against class A, class C, and some class D β-lactamases [41]. Avibactam combined with ceftazidime has been reported to restore its activity against CRE isolates carrying KPC and OXA-48 carbapenemases [42], but it is not active against MBL producing strains [43]. For example, ceftazidime–avibactam combination showed 100% susceptibility in CRE KPC and OXA-48 producers. In addition, imipenem–relebactam has demonstrated good activity against carbapenem-resistant isolates, specifically those that produce KPC enzymes [41]. Given the clinical availability of novel β-lactamase inhibitors that specifically target subsets of carbapenemases, the reliable classification of the carbapenemase class can assist in immediate therapeutic implications.

Regarding the performance of the BD CPO detect panel in assigning carbapenemases to three Ambler classes, previous studies have reported varying sensitivity [34,38]. In this study, the BD Phoenix CPO detect panel correctly assigned Ambler class carbapenemases to 67.0% (*n* = 73/109) CPE isolates. Our Ambler classification results comply partly with Simon and co-workers [38] and Cho and co-workers [34], who revealed a sensitivity of 79.0% (*n* = 45/57) and 83.0% (*n* = 39/47), respectively, for Ambler typing of CPE isolates. However, the diversity of carbapenemase types in previous studies was different, reflecting the local current epidemiology of carbapenemase. The differences observed in the performance of Ambler classification between the current study and previous studies could be due to the variations in the number of CPE isolates tested. Moreover, the differences in carbapenemase types and classes among the three study strain collections could account for the observed variation. We tested almost double the number of CPE isolates compared to previous studies.

The panel identified and categorized Ambler classes A, B, and D to 78.8%, 65.9%, and 56.3% CPE isolates correctly. The highest sensitivity was observed in detecting class A KPC producing isolates, while the lowest sensitivity was observed in detecting class D OXA-48-like isolates. Detecting class D OXA-48-like enzymes is notoriously challenging because they often cause low-level in vitro resistance to carbapenems [44]. In this study, *K. pneumoniae (n* = 48) was the most prevalent CPE isolate, and the BD CPO detect panel correctly identified 81.3% (*n* = 13/16) of *K. pneumoniae* KPC isolates and 75% (*n* = 15/20) of *K. pneumoniae* OXA-48-like isolates as harboring class A and class D carbapenemase, respectively. However, one *K. pneumoniae* strain with class A KPC and one with class D OXA-48-like were misclassified as a class B carbapenemase producer. Furthermore, two class A and four class D *K. pneumoniae* strains remained unclassified for the Ambler class. KPC and OXA-48-like are the most clinically important carbapenemases among CPO *K. pneumoniae* in Europe and Korea, respectively [45,46]. *K. pneumoniae* strains of OXA-48-like CPE are common in Korea [46]. Jonas and co-workers found that the BD CPO detect panel classified 27.1% (*n* = 62/229) of *K. pneumoniae* KPC isolates from a European collection as carrying class A carbapenemase [47]. Additionally, they reported the sensitivity of the CPO detect panel in assigning Ambler class B and D to *K. pneumoniae* was 86% and 91%, respectively [47]. We observed some limitations of the CPO panel in assigning Ambler class B to CPE isolates (31.8%), where the panel failed to assign 40.0% IMP and 63.7% VIM isolates to the corresponding Ambler class. Moreover, 34.4% (*n* = 11/32) of class D OXA-48-like CRE strains were not assigned to Ambler class, although the CPO detect panel identified 96.9% (*n* = 31/32) strains to be positive for carbapenemase. In this study, almost one-third (*n* = 30/109) of CPE isolates were unclassified for Ambler class A, class B, or class D by the BD CPO detect panel.

We found one VIM class B carbapenemase producing *K. oxytoca* strain with discrepancies in the identification of carbapenemase class and carbapenem susceptibility pattern. The automated platforms may cause discrepancies in the detection of carbapenemase producers [48]. However, compared to manual tests for detecting CPOs, the BD CPO detect panel has several advantages, such as reduced hands-on time, operator independence, and providing carbapenemases Ambler classes at the same time as the results of initial phenotypic AST.

The advantage of this study is that we evaluated a large number of CPE clinical isolates with resistance gene characteristics, particularly *K. pneumoniae* (*n* = 46). Currently, *K. pneumoniae* is the dominant CRE strain in Korea due to its ability to easily colonize and rapidly spread in hospitals [10]. In 2019, *K. pneumoniae* comprised 59.9% of the 8333 CRE isolates analyzed by the Research Institute of Public Health and the Environment (RIPHE), Korea [10]. We think the correct identification of *K. pneumoniae* CPE possessing KPC and OXA-48-like resistance genes might be important in Korea considering the local prevalence of CPE.

This study has some limitations. First, the inclusion of only CPE clinical isolates and not the non-CPE isolates in this study; however, the CPO detect panel was evaluated against a large collection of genotypically characterized multicenter CPE clinical isolates with different resistance patterns. Although we did not compare CRE and non-CRE, this study showed the reliability of the CPO detect panel against class A carbapenemase producing CRE. Second, there are not enough IMP (*n* = 10) and VIM (*n* = 11) carbapenemase producing strains among the CPE isolates tested. This is primarily because these two carbapenemase types are rare in Korea [10]. A total of 9 VIM-1 type CRE isolates were reported in Korea by the RIPHE in 2019 [10]. Finally, we did not investigate the molecular basis for the discrepancy in detecting *K. oxytoca* as a class B carbapenemase and ESBL producer, as well as its carbapenem susceptibility as tested by the BD CPO detect panel. However, carbapenem susceptibility testing by repeated BD CPO detect panel and MicroScan method was performed to rule out the handling error.

In conclusion, the BD Phoenix CPO detect panel is a sensitive test for detecting CPE and classifying *K. pneumoniae* producing KPC as Ambler class A. However, we found that the CPO detect panel correctly classified only 78.8%, 65.9%, and 56.3% carbapenemase producers to Ambler class A, class B, and class D, respectively. The panel had a low sensitivity for the detection of class B VIM and class D OXA-48-like carbapenemase producing isolates. Its sensitivity needs to be improved for the Ambler classification, particularly for the class B and class D carbapenemase producers.

## 4. Materials and Methods

### 4.1. Bacteria Strains

A total of 109 *Enterobacterales* clinical isolates collected from routine clinical samples processed at 5 different hospitals in Seoul, Korea from January 2016 to December 2018 were included in this study. These include 48 *K. pneumoniae*, 19 *E. cloacae*, 15 *C. freundii*, 12 *E. coli*, 9 *K. oxytoca*, 3 *E. aerogenes*, and 3 *S. marcescens* isolates. Duplicate isolates from the same patient were excluded from the study to avoid redundancy. The resistance of bacterial isolates to carbapenemases was previously confirmed using the MicroScan WalkAway 96 plus system (Beckman Coulter, Atlanta, GA, USA). All the strains were resistant to at least one of the carbapenem antibiotics (imipenem, meropenem, ertapenem, and doripenem).

PCR for carbapenemases genes (*blaKPC, blaNDM, blaVIM, blaIMP*, and *blaOXA-48-like*) was performed in a hospital and Sanger sequencing was performed by Macrogen (Seoul, Republic of Korea) to determine the genotype of *Enterobacterales* isolates. All isolates were PCR positive for the carbapenemase gene (CPE). Strains were identified by matrix-assisted laser desorption ionization/time-of-flight mass spectrometry (MALDI-TOF MS; Bruker Microflex LT, Bruker Daltonik GmbH, Bremen, Germany). The stock cultures of isolates were stored in skimmed milk at −70 °C and subcultured on blood agar plates (BAP) for further use. The performance of the BD CPO detect panel was evaluated at a single center.

### 4.2. BD Phoenix CPO Detect Test

The BD Phoenix CPO detect panel was used to detect and classify carbapenemase in CPE isolates according to the manufacturer’s instructions (BD Diagnostic Systems, Sparks, MD, USA). Briefly, the CPE isolates were subcultured on blood agar plates (BAP) overnight at 37 °C. The overnight grown colonies were suspended in Phoenix identification (ID) broth at a concentration of 0.5 McFarland standard. The standardized ID broth bacterial suspension (25 μL) was then transferred into the Phoenix AST broth, which was supplemented with one drop of Phoenix AST indicator (methylene blue and resazurin) for detecting bacterial growth and then poured into the BD Phoenix™ NMIC-500 panels (BD Catalogue no. 449023). The panels were sealed, logged, and inserted into the BD Phoenix™ M50 instrument. The results were analyzed using Epicenter data management software (v. 6.61A, BD Diagnostic Systems) after 6–16 h of incubation. The CPO detect panel was subjected to a quality control check according to the manufacturer’s instructions. The isolates identified as carbapenemase producers by the CPO detect panel were assigned to the Ambler class or remained unclassified.

The accuracy of the BD Phoenix NMIC-500 panel in detecting carbapenemase activity (P/N) and classifying carbapenemase into Ambler class A, class B, and class D in CPE clinical isolates was determined by comparing to the presence of carbapenemase genes characterized by PCR.

## Figures and Tables

**Table 1:** Performance of the BD Phoenix CPO detect panel for carbapenemase detection and Ambler classification in CPE clinical isolates.

		BD Phoenix CPO Detect Panel						
Carbapenemase Class and Type Species	Isolates Tested (*n*)	Carbapenemase Production (P/N)		Carbapenemase Ambler Classification				
		Positive	Negative	Correct	A	B	D	Unclassified Positive
Class A	33	33	-	26	26	2	-	5
KPC	33							
*C. freundii*	2	2	-	1	1	1	-	-
*E. coli*	6	6	-	5	5	-	-	1
*E. aerogenes*	2	2	-	2	2	-	-	-
*E. cloacae*	3	3	-	3	3	-	-	-
*K. oxytoca*	1	1	-	1	1	-	-	-
*K. pneumoniae*	16	16	-	13	13	1	-	2
*S. marcescens*	3	3	-	1	1	-	-	2
Class B	44	43	-	29	-	29	-	14
IMP	10							
*C. freundii*	2	2	-	2	-	2	0	0
*E. cloacae*	5	5	-	3	-	3	0	2
*K. pneumoniae*	3	3	-	1	-	1	0	2
NDM	23							
*C. freundii*	6	6	-	6	-	6	0	0
*E. coli*	2	2	-	2	-	2	0	0
*E. cloacae*	8	8	-	6	-	6	0	2
*K. oxytoca*	2	2	-	2	-	2	0	0
*K. pneumoniae*	5	5	-	4	-	4	0	1
VIM	11							
*C. freundii*	4	4	-	3	-	3	0	1
*E. cloacae*	1	1	-	-	-	-	-	1
K. oxytoca *	2	1	1	-	-	-	-	1
*K. pneumoniae*	4	4	-	-	-	-	-	4
Class D	32	31	1	18	-	2	18	11
OXA-48-like	32							
*C. freundii ^#^*	1	-	1	-	-	-	-	-
*E. coli*	4	4	-	2	-	-	2	2
*E. aerogenes*	1	1	-	1	-	-	1	-
*E. cloacae*	2	2	-	-	-	-	-	2
*K. oxytoca*	4	4	-	-	-	1	-	3
*K. pneumoniae*	20	20	-	15	-	1	15	4

*, *K. oxytoca* producing VIM class B carbapenemase was identified as carbapenemase negative and ESBL producer by routine BD CPO detect panel test; however, duplicated tests showed class B carbapenemase and ESBL producer (Table S1). *K. oxytoca* also showed a discrepancy in the carbapenem susceptibility testing by repeated BD CPO detect panel and MicroScan method (Table S2). It harbors *blaVIM* gene by PCR. ^#^, *C. freundii* producing OXA-48-like class D carbapenemase was detected as carbapenemase negative by routine BD CPO detect panel test and repeated subsequent tests also showed carbapenemase negative (Table S1). It harbors *blaOXA-48-like* gene by PCR. KPC, *Klebsiella pneumoniae* carbapenemase; NDM, New Delhi metallo-β-lactamase; IMP, imipenemase; VIM, Verona integron-born metallo-β-lactamase; OXA, oxacillinase.

## Data Availability

The data presented in this study are available in the article or supplementary material.

## Supplementary Materials

The following supporting information can be downloaded at: https://www.mdpi.com/article/10.3390/antibiotics12071215/s1, Table S1: Carbapenemase detection and Ambler classification by BD Phoenix CPO detect panel; Table S2: Carbapenemase susceptibility testing by BD Phoenix CPO detect panel and MicroScan.
