# Supplementary material for: Evaluation of the BD Phoenix CPO Detect Panel for Detection and Classification of Carbapenemase Producing Enterobacterales
Source: Antibiotics (Basel). 2023 Jul 21;12(7):1215. doi: 10.3390/antibiotics12071215 (PMC10376851; doi:10.3390/antibiotics12071215)
Supplement: Supplementary file 1 [file antibiotics-12-01215-s001.zip › antibiotics-2461548-supplementary.pdf]

antibiotics

Supplementary Materials

Table S1. Carbapenemase detection and Ambler classification by BD Phoenix CPO detect panel.

Strain ID	CP Gene (PCR)	Bacterial Species	CPO Detect Panel (#1)	CPO Detect Panel (#2)	CPO Detect Panel (#3)
HL 848	*VIM*	*Klebsiella oxytoca*	Extended Spectrum Beta-lactamase		Class B Carbapenemase Producer, Extended Spectrum Beta-lactamase
HL 1070	*OXA-48-like*	*Citrobacter freundii*	No CRE	No CRE	No CRE

CRE, Carbapenem-resistant *Enterobacterales*.

Table S2. Carbapenemase susceptibility testing by BD Phoenix CPO detect panel and MicroScan.

Strain ID	CP Gene (PCR)	Bacterial Species	CPO Detect Panel (#1)			CPO Detect Panel (#2)		
			Ertapenem	Imipenem	Meropenem	Ertapenem	Imipenem	Meropenem
HL 848	*VIM*	*Klebsiella oxytoca*	>1, R	2, I	0.5, S	>1, R	4, R	2, I
HL 1070	*OXA-48-like*	*Citrobacter freundii*	0.5, S	2, I	≤0.25, S	≤0.25, S	2, I	≤0.25, S
			**MicroScan (#1)**			**MicroScan (#2)**		
			Ertapenem	Imipenem	Meropenem	Ertapenem	Imipenem	Meropenem
HL 1070	*OXA-48-like*	*Klebsiella oxytoca*	0.5, S	1, S	≤0.25, S	1, I	2, I	≤1, S

S, susceptible; I, intermediate; R, resistant.
